# A Fluorine-Containing Main-Chain Benzoxazine/Epoxy Co-Curing System with Low Dielectric Constants and High Thermal Stability

**DOI:** 10.3390/polym15234487

**Published:** 2023-11-22

**Authors:** Tinghao Zhang, Wenzheng Zhang, Zichun Ding, Jianjian Jiao, Jinyan Wang, Wei Zhang

**Affiliations:** 1 Shenyang Special Rubber Basic Research Key Laboratory, Shenyang University of Chemical Technology, 11th Street, Shenyang Economic and Technological Development Zone, Shenyang 110142, China; 19818965934@163.com; 2 State Key Laboratory of Fine Chemicals, Liaoning High Performance Resin Engineering Research Center, Department of Polymer Science and Engineering, Dalian University of Technology, Dalian 116024, China; dzc3288@163.com (Z.D.); wangjinyan@dlut.edu.cn (J.W.); 3 China-Spain Joint Laboratory on Material Science, Shenyang University of Chemical Technology, Shenyang Economic and Technological Development Zone, Shenyang 110142, China; jiaojj_ly@163.com; 4 Department of Technoloy, Xi’an Aibang Electromagnetic Technology Co., Ltd., Building B4, Biyuan Second Road Artificial Intelligence Industrial Park, Chang’an District, Xi’an 710199, China; wzhang1980@163.com

**Keywords:** main-chain benzoxazine, copolymer, high-performance composite, dielectric property

## Abstract

A fluorine-containing main-chain benzoxazine (BAF-M-TB) was co-cured with biphenyl epoxy for the integrated circuit industry. The benzoxazine precursor was synthesized using 4,4′-(Hexafluoroisopropylidene)diphenol (bisphenol AF), 2,2′-Dimethyl-[1,1′-biphenyl]-4,4′-Diamine(M-TB), and paraformaldehyde. In addition, the 3,3′-(Oxybis(4,1-phenylene))bis(3,4-dihydro-2H-benzo[e][1,3]oxazine) (Benoxazine ODA-BOZ), which is a commercialized benzoxazine, was co-cured with biphenyl epoxy as a control. The two co-curing systems were referred to as EP/BAF-M-TB and EP/ODA-BOZ. The curing kinetics, rheological behavior, and thermal stability of the two co-curing systems were studied. Poly-EP/BAF-M-TB and poly-EP/ODA-BOZ quartz fiber cloth reinforced composites (QFRPs) were prepared using the prepreg laminating method in order to determine their mechanical, thermal, and dielectric properties. Both of them showed good thermal properties and dielectric properties. The dielectric constant of poly-EP/BAF-M-TB QFRP is in the range of 3.25–3.54 at the low frequency of 10 kHz–10 MHz. At the high frequency of 5 GHz, its dielectric constant is 3.16, which is better than that of poly-EP/ODA-BOZ QFRP. Additionally, the Td_5_ of poly-EP/BAF-M-TB was 398 °C in a nitrogen atmosphere, which is higher than that of poly-EP/ODA-BOZ.

## 1. Introduction

With the development of science and technology, composite materials have come to play an indispensable role in human society [1,2]. With thermosetting resin as the prepreg, quartz fiber cloth reinforced plastics (QFRPs) with a low dielectric constant, low dielectric loss, and excellent thermal properties have gradually become the favored option in the integrated circuit industry [3]. This is because the reduction in the dielectric constant and dielectric loss can, respectively, enhance the transmission speed of the signal and minimize the loss of the signal during transmission. As a thermosetting resin, benzoxazine has many excellent properties, including a high glass transition temperature (T_g_) and low dielectric constants and dielectric losses [4,5,6,7,8]. Due to these advantages, benzoxazine is considered to be an ideal material for prepreg [9]. The cross-linking density refers to the number of cross-linking points in a resin, which significantly impacts its mechanical and thermal properties. Meanwhile, they possess a high modulus, and T_g_, benzoxazines exhibit a relatively low cross-linking density. By further tightening the network structure, higher-performance thermosetting resins can be achieved. Hydrogen bonding is a weak interaction. In benzoxazine, it limits the mobility of the segment and prevents the formation of the network [10,11,12].

It is feasible to introduce epoxy for co-curing to tighten its network structure by introducing extra cross-linking points. Epoxy resin (EP) has been widely used in adhesives and coatings, copper-clad plating, and other industries [13]. The epoxy groups can be cured using phenolic curing agents under heating conditions, and they can usually be self-polymerized by alcohol hydroxyl groups via ring opening. The ring-opening polymerization of benzoxazine can produce a phenolic hydroxyl group and tertiary amines. Because the phenolic hydroxyl group is more active than the alcohol hydroxyl group, the epoxy group normally only reacts with the phenolic hydroxyl group under the catalysis of tertiary amines [14]. Therefore, it is feasible to co-cure benzoxazine with epoxy resin to achieve a higher crosslinking density. XU et al. blended the benzoxazine BA-Ph with the epoxy resin DEGBA and made it into a glass fiber composite material. Compared with BA-Ph-based GFRP, the BA-Ph/EP-based GFRP shows better mechanical and thermal properties [15]. Some cases were also reported earlier [16,17]. Overall, the existing EP/benzoxazine copolymerization systems all show impressive thermal and mechanical properties. However, the choice of benoxazines to use in the co-curing systems comprises the monofunctional or bifunctional types. The use of main-chain-type polybenzoxazine precursors is rarely reported.

Main-chain-type polybenzoxazine precursors, which contain benzoxazine groups as repeating units in the main chain, have been widely studied [18,19,20,21,22,23]. Gu et al. [20] synthesized a main-chain-type polybenzoxazine precursor using bisphenol-A, 4,4′-diaminodiphenylmethane, aniline, and paraformaldehyde. Compared with bifunctional benzoxazine synthesized using bisphenol-A, aniline, and paraformaldehyde [24], this kind of main-chain benzoxazine has lower dielectric losses and dielectric constants. Lin et al. [22] synthesized a series of main-chain benzoxazine with different molecular weights. The dielectric properties and thermal stability of the cured benzoxazine increased with the increase in the molecular weight. Moreover, the introduction of a C-F bond in the polymer repeat units was proven to be an effective means of reducing the dielectric constant and dielectric loss [25,26]. In addition, the introduction of trifluoromethyl also improves the thermal stability due to the high binding energy of the C-F bond [27,28]. There are more cases of introducing a C-F bond into bifunctional benzoxazines [29,30], and fewer cases of introducing a C-F bond into main-chain benzoxazines. Herrera et al. [31] synthesized a series of high-fluorinated main-chain benzoxazine polymers by combining high-fluorinated diamine with 4,4′-(Hexafluoroisopropylidene) diphenol (bisphenol AF). A series of similar hydrogenated versions were synthesized as control groups. The experimental results show that the main-chain-type benzoxazines containing C-F bonds have higher thermogravimetric temperatures and better dielectric constants than the hydrogenated main-chain-type benzoxazines. The synthesis of high-fluorinated diamine is relatively challenging, and it has difficulty meeting the needs of industrial production. Therefore, it is still sensible to choose to use bisphenol AF to introduce trifluoromethyl groups into benzoxazines. In general, it is important to prepare the co-curing resin system of epoxy and fluorine-containing main-chain benzoxazine and apply it to quartz fiber composites. However, similar works have rarely been reported.

In this study, 2,2′-Dimethyl-[1,1′-biphenyl]-4,4′-Diamine(M-TB), bisphenol AF, and paraformaldehyde were used to prepare the main-chain-type benzoxazine precursor, which was referred to as BAF-M-TB. The molecular structure of the benzoxazine precursor was characterized. In addition, 3,3′-(oxybis(4,1-phenylene))bis(3,4-dihydro-2H-benzo[e][1,3]oxazine) (ODA-BOZ), a benzoxazine already industrialized in the copper cladding industry, was used as a control. Then, two benzoxazine precursors were blended with 4,4′-bis(oxiran-2-yloxy)-1,1′-biphenyl (YX4000), and their curing behavior, curing kinetics, and rheological processing were studied. At the same time, the thermal stability of the cured samples was investigated. The QFRPs were prepared from the blended prepolymers. The thermal, mechanical, and dielectric properties of the composite samples were investigated. The obtained poly-EP/BAF-M-TB QFRP showed excellent dielectric properties and expanded the design premise and application scenario of main-chain benzoxazine.

## 2. Materials and Methods

### 2.1. Materials

Bisphenol AF (AR), paraformaldehyde (AR), n-hexane (99.5%), and sodium hydroxide (97%) were purchased from Macklin Biochemical Co., Ltd., Shanghai, China. M-TB (99%) was purchased from Bide Pharmatech Co., Ltd., Shanghai, China. N, N-Dimethylformamide (DMF) (AR) was obtained from Aladdin Biochemical Technology Co., Ltd., Shanghai, China. Xylene (AR) was obtained from Nanjing chemical reagent Co., Ltd., Nanjing, China. All chemicals were used directly as received. An epoxy resin (YX4000) was obtained from Taiji new material Co., Ltd., Guangzhou, China. The resin is a pale yellow powder at room temperature. ODA-BOZ (BZ3100) resin was obtained from Enying Polymer Materials Co., Ltd., Puyang, China. The resin is a brown-red tablet at room temperature. High-strength quartz fiber cloth (SJ108), with a thickness of 0.18 mm and a specific weight of 180 g/m^2^, was obtained from Shenjiu Tianhang new materials Co., Ltd., Zhengzhou, China.

### 2.2. Synthesis of BAF-M-TB

**Scheme 1:**
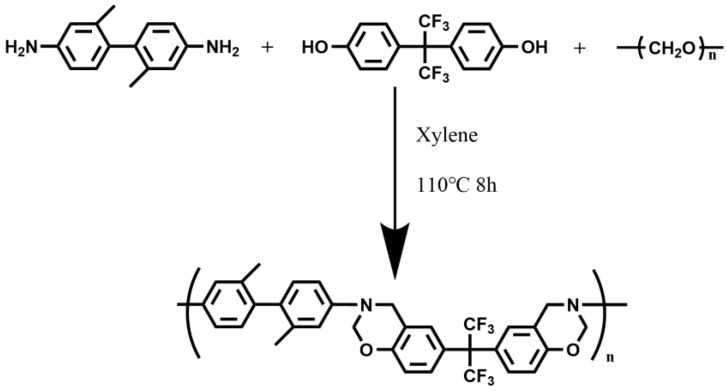
Synthesis of the BAF-M-TB precursor.

The molar ratio of the amine, phenol hydroxyl, and aldehyde groups used in the synthesis of BAF-M-TB was determined to be 1:1:3. A mixture of bisphenol AF, M-TB, and paraformaldehyde was dissolved into xylene and then reflowed for 8 h at 110 °C in a three-way flask fitted with a condenser; the reaction mechanism is shown in Scheme 1. The resulting crude product was a yellow liquid, and *n*-hexane was used to dissolve the solvent. Then, it was soaked in 1N aqueous sodium hydroxide solution to remove all phenols. The mixture was then cleaned to neutral using deionized water. Then, the crude product was freeze-dried. The beige target product was obtained.

### 2.3. Preparation of the EP/BAF-M-TB and EP/ODA-BOZ Blend

The EP/BAF-M-TB blend was prepared as follows. First, a measured weight of the EP monomer and BAF-M-TB was added to DMF. The molar ratio of the epoxy group and oxazine group was 1/1, while the mass of the solid and the solvent was controlled as 1/10. Second, the above mixture was refluxed at 150 °C for 120 min, and a yellow prepolymer solution was obtained. The EP/ODA-BOZ blend was prepared using the same method, and a black prepolymer solution was obtained.

### 2.4. Preparation of EP/BAF-M-TB QFRP Composites

QC (quartz fiber cloth) fabric (10 cm × 6 cm) was infiltrated with the EP/BAF-M-TB prepolymer solution and dried at 140 °C for 30 min. The EP/BAF-M-TB blend QC prepreg was obtained. The ratio was designed to produce a prepreg of 50 wt% blend and 50 wt% QC, theoretically. Ten layers of prepreg were heated at 170 °C for 20 min which was in order to complete melted it and then hot-pressed under a pressure of 2.5 MPa with a procedure according to processability and curing behavior studies (200 °C/3 h; 240 °C/3 h; 270 °C/2 h; 290 °C/1 h, respectively). After gradually cooling to room temperature, the black EP/BAF-M-TB composite laminates were obtained.

### 2.5. Preparation of EP/ODA-BOZ QFRP Composites

QC fabric (10 cm × 6 cm) was infiltrated with EP/BAF-M-TB prepolymer solution and dried at 140 °C for 30 min. The EP/BAF-M-TB blend QC prepreg was obtained. The ratio was designed to give a prepreg of 50 wt% blend and 50 wt% QC, theoretically. Ten layers of prepreg were heated at 140 °C for 20 min to full melting and were then hot-pressed under a pressure of 2 MPa following a procedure according to processability and curing behavior studies (170 °C/3 h; 225 °C/3 h; 265 °C/2 h). After gradually cooling to room temperature, the black EP/ODA-BOZ composite laminates were obtained.

### 2.6. Representation

#### 2.6.1. Fourier Transform Infrared Spectroscopy (FT-IR)

The Fourier Transform Infrared Spectroscopy was carried out on a Nicolet iS50 FT-IR (Thermo Scientific, Waltham, MA, USA). The sample was processed using the KBr-disk method. The sample recorded FT-IR spectra within the wave numbers of 500–4000 cm^−1^.

#### 2.6.2. Nuclear Magnetic Resonance (NMR)

^1^H nuclear magnetic resonance spectra (^1^H NMR) were obtained using an Avance neo 500 m NMR (Bruker, Zurich, Switzerland), and ^13^C NMR and ^19^F NMR spectra were obtained using an ASCEND 600 MHz Bruker instrument (Switzerland). The test specimen was dissolved in (Methyl sulfoxide)-d6 (DMSO-d6).

#### 2.6.3. Size Exclusion Chromatography (SEC)

Size exclusion chromatographic measurements were taken using a 2695 high-performance liquid chromatography instrument (Waters, Milford, CT, USA). *N*-methylpyrrolidinone (NMP) was used as a carrier solvent at a flow rate of 1.0 mL/min.

#### 2.6.4. Differential Scanning Calorimetry (DSC)

Differential scanning calorimetry was performed using a DSC 3+ (Mettler Toledo, Zurich, Switzerland), and measurements were recorded at various heating rates under a nitrogen atmosphere. Experiments were always performed below 350 °C to prevent any possible degradation reactions inside the chamber. The samples were weighted after each non-isothermal test, and there was no obvious weight loss.

#### 2.6.5. Dynamic Rheological Analysis

The dynamic rheological analysis (temperature and time scan) was performed using an AR-G2 Rheometer (TA Instruments, New Castle, DE, USA) with a frequency of 1 Hz at different temperatures in air. The samples (0.5–0.6 g) were melted between 25 mm diameter parallel plates with the environmental testing chamber of the rheometer.

#### 2.6.6. Dynamic Thermal Mechanical Analysis (DMA)

The dynamic thermal mechanical analysis was performed using a Q800 DMA (TA Instruments, New Castle, USA) with a single cantilever beam model. The sample (length: 45 mm, width: 7 mm, thickness: approximately 2 mm) was measured with a drive frequency of 1 Hz and a heating rate of 5 °C/min ranging from 30 to 400 °C.

#### 2.6.7. Thermogravimetric Analysis (TGA)

Thermogravimetric analysis was performed using a Q500 TGA (TA Instruments, New Castle, USA) with a heating rate of 10 °C/min in a nitrogen atmosphere. Dynamic thermogravimetric (DTG) data were also recorded simultaneously.

#### 2.6.8. Flexural Test

Flexural tests (three-point bending mode) were performed using a 5848 MicroTester (Instron, Boston, MA, USA) according to the GB/T 9341-2008/ISO 178 standard test method with a crosshead displacement speed of 10 mm/min; the test fixture was mounted in a 10 kN capacity [32]. The samples (length: 80 mm, width: 15 mm, thickness: approximately 2 mm) were tested with a support span/sample thickness ratio of 15:1 and gained an average value for every five samples.

#### 2.6.9. Dielectric Property Measurement

Dielectric properties in low frequencies were tested using a Keysight 4294A (Agilent, Santa Clara, CA, USA) precision impedance analyzer in a frequency range of 10 kHZ–10 MHz. The samples (length: 20 mm, width: 20 mm, thickness: approximately 0.8 mm) were dried before measurements and then tested at room temperature.

Dielectric properties at high frequencies were tested using a Keysight N5232A (Agilent, Santa Clara, CA, USA) microwave vector network analyzer using the split-post method at microwave frequencies of 5 GHz. The samples (length: 60 mm, width: 40 mm, thickness: approximately 0.8 mm) were dried before measurements were taken and then tested at room temperature.

## 3. Results and Discussion

### 3.1. Chemical Structural Characterization of BAF-M-TB

**Figure 1:**
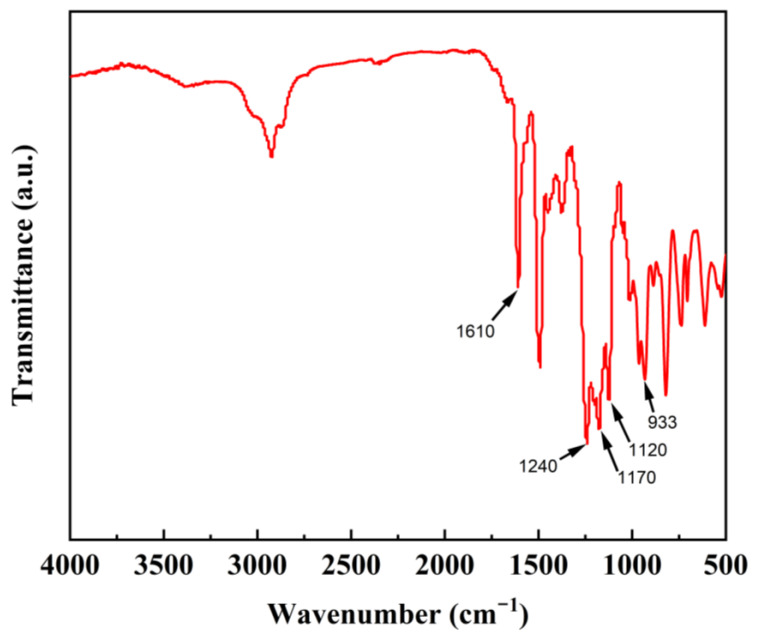
The FT-IR spectra of the BAF-M-TB precursor.

The FT-IR spectra of the BAF-M-TB precursor are shown in Figure 1. The absorption of benzene with an attached oxazine ring is located at 933 cm^−1^, the absorption of the C-O-C stretching mode is located at 1240 cm^−1^, and the absorption of the C-N-C stretching mode is located at 1170 cm^−1^. The absorption of in-plane carbon–carbon stretching of the trisubstituted benzene ring was located at 1610 cm^−1^. Moreover, the absorption peak at 1121 cm^−1^ was attributed to C-F in trifluoromethyl. These absorptions display important information, suggesting that BAF-M-TB benzoxazine resin was successfully obtained.

**Figure 2:**
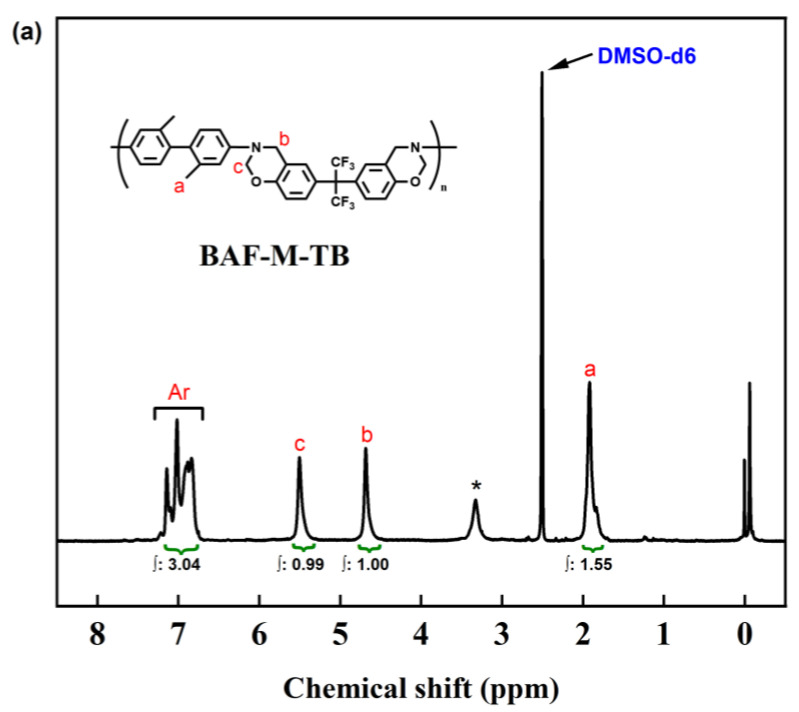

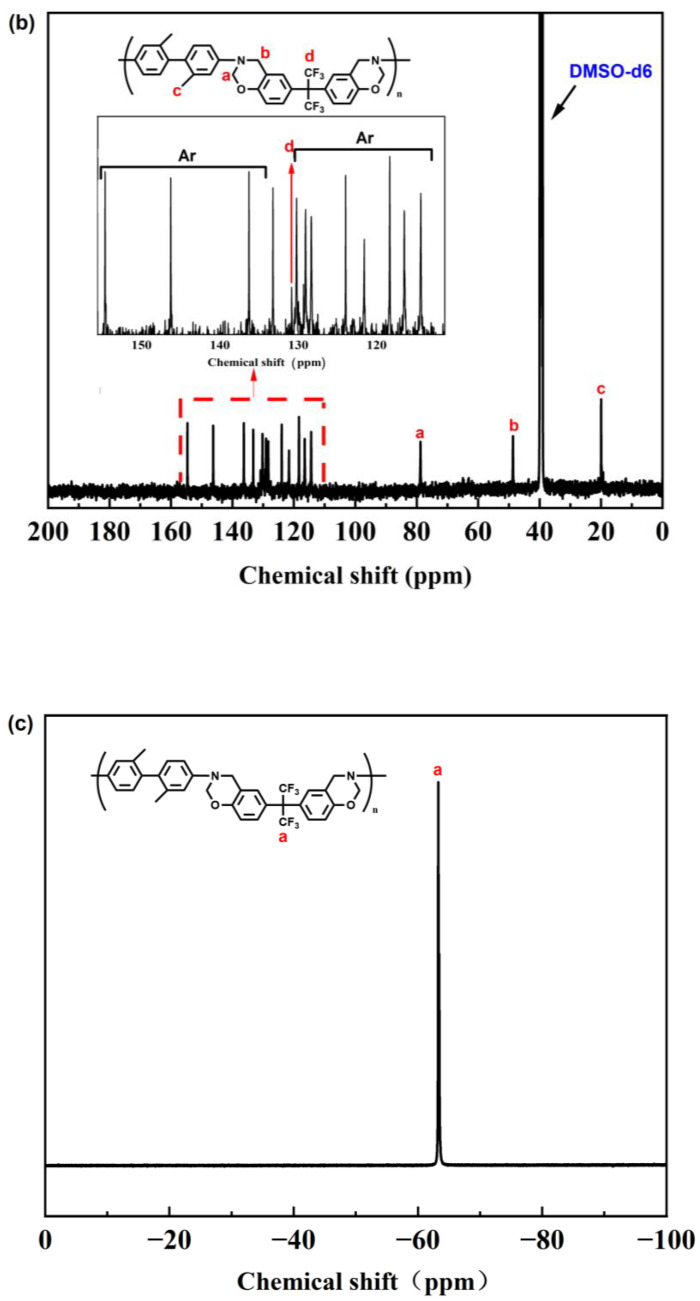
(**a**) The ^1^H-NMR spectra of the BAF-M-TB precursor. (**b**) The ^13^C-NMR spectra of the BAF-M-TB precursor. (**c**) The ^19^F-NMR spectra of the BAF-M-TB precursor.

To further confirm the structure of the BAF-M-TB precursor, ^1^H-NMR, ^13^C-NMR, and ^19^F-NMR spectra, as shown in Figure 2, were used. The BAF-M-TB precursor has two different -CH_2_-groups on the oxazine ring. The ^1^H-NMR spectra show that the first resonance peak appears at 4.69 ppm, corresponding to the chemical structure of Ar-CH_2_-N-, and the second resonance peak appear at 5.50 ppm, corresponding to the chemical structure of -O-CH_2_-N-. In addition, the resonance peak at 1.55 ppm was attributed to the methyl group on the biphenyl structure; the * in the Figure 2a represents the resonance peak of water. The ^13^C-NMR spectra show that the signal peaks for the chemical structure of Ar-CH_2_-N- on the oxazine rings appear at 48.7 ppm, and the signal peaks for the chemical structure of -O-CH_2_-N- on the oxazine rings appear at 78.9 ppm. In addition, the signal peak of the methyl group on the biphenyl structure is 19.9 ppm; the trifluoromethyl signal peak is located at 130.8 ppm. Finally, the trifluoromethyl signal peak at −63.2 ppm was observed by the ^19^F-NMR spectra. The results of NMRs and FI-TR indicate that BAF-M-TB precursor was synthesized.

The molecular weight and polydispersity (PDI) of the BAF-M-TB precursor were measured using SEC, and the molecular weight distribution curves are shown in Figure S1. The M_n_ of the oligomers was estimated to be 4540 Da, and the M_w_ of the oligomers was estimated to be 12,797 Da, with a PDI value of 2.82. The measured molecular weight showed that the precursor is mainly composed of heptamers and octamers.

### 3.2. Curing, Rheological Behavior, and Processing of the Copolymer

**Figure 3:**
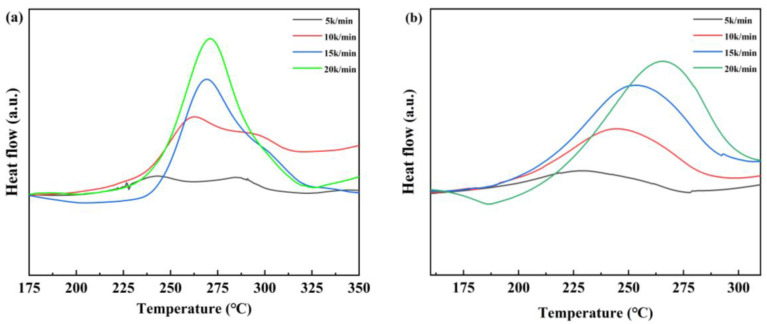
DSC thermograms of (**a**) EP/BAF-M-TB and (**b**) EP/ODA-BOZ.

**Figure 4:**
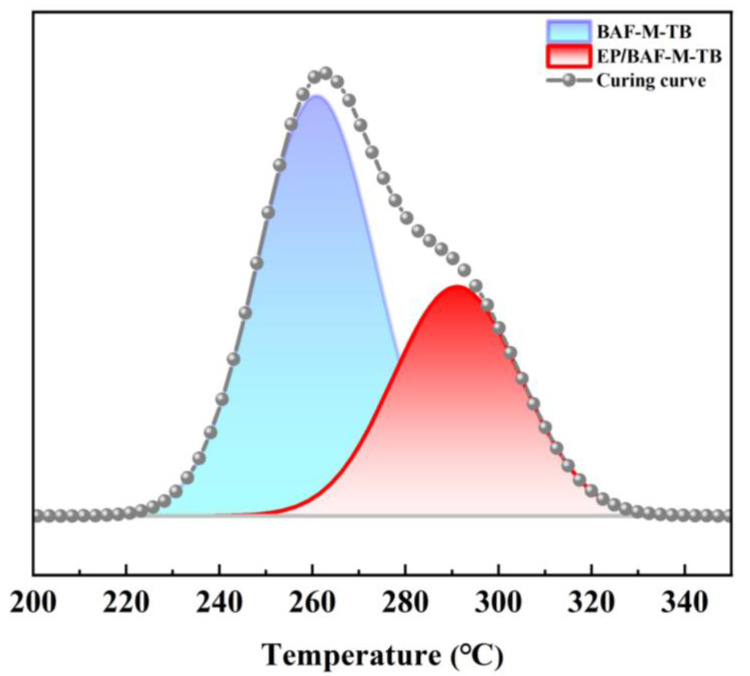
The two-stage curing exothermic process of EP/BAF-M-TB.

DSC thermograms of EP/BAF-M-TB and EP/ODA-BOZ heating at different rates are shown in Figure 3. The exothermic process of the EP/BAF-M-TB prepolymer clearly has two stages, and the exothermic peak is divided into two using the Gaussian method, as shown in Figure 4. The starting, maximum, and ending positions of the exothermic peaks of the EP/BAF-M-TB prepolymer and the EP/ODA-BOZ prepolymer are defined as the T_i_, T_p_, and T_d_ temperatures, respectively. The exothermic peaks shifted to higher temperatures with an increase in the heating rate because of the thermal hysteresis activity. The exothermic peaks’ temperature increased from 243.8 °C to 270.8 °C for the first-stage curing process and from 283.3 °C to 302.1 °C for the second-stage curing process. With these curing process parameters, the activation energy (E_a_) was calculated using the Kissinger [33] (Equation (1)) and Ozawa [34] (Equation (2)) methods:

lnβ/Tp = lnAR/E_a_ − E_a_/RT_p_ (1)

**Figure 5:**
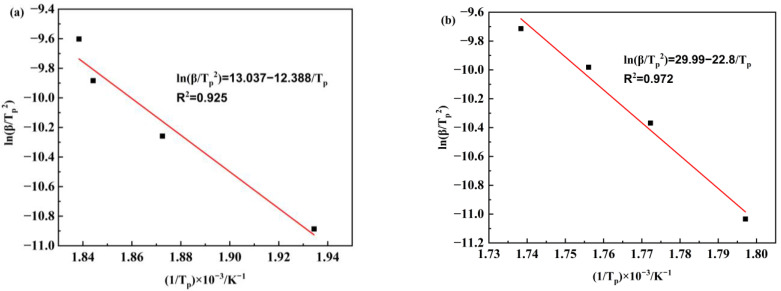
(**a**) Calculation of the first-peak E_a_ values of EP/BAF-M-TB prepolymer using the Kissinger method. (**b**) Calculation of the second-peak E_a_ values of EP/BAF-M-TB prepolymer using the Kissinger method.

**Figure 6:**
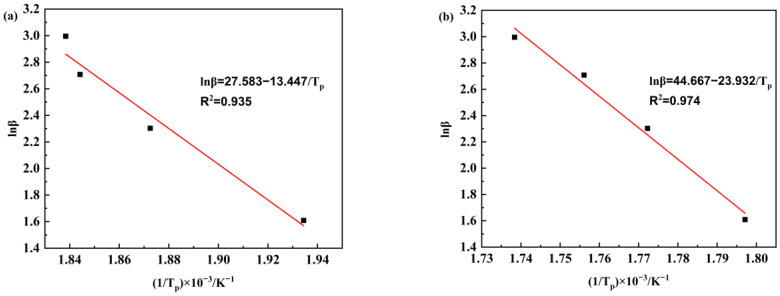
(**a**) Calculation of the first-peak E_a_ values of the EP/BAF-M-TB prepolymer using the Ozawa method. (**b**) Calculation of the second-peak E_a_ values of the EP/BAF-M-TB prepolymer using the Ozawa method.

**Figure 7:**
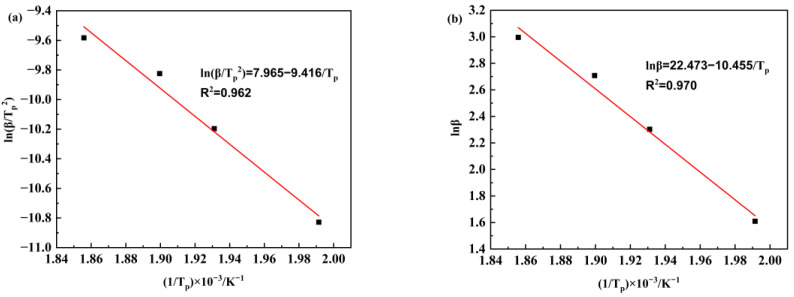
(**a**) Calculation of the first-peak E_a_ values of the EP/ODA-BOZ prepolymer using the Kissinger method. (**b**) Calculation of the first-peak E_a_ values of the EP/ODA-BOZ prepolymer using the Ozawa method.

Lnβ = −1.052E_a_/RT_p_ + C (2)

where E_a_ represents the activation energy and β, A, and R represent the heating rate, Arrhenius constant, and ideal gas constant, respectively. The curing process parameters of the exothermal peaks were put into the equations. Meanwhile, ln(β/T_p_^2^) and 1/T_p_ of EP/BAF-M-TB were plotted (Figure 5), lnβ and 1/T_p_ were plotted (Figure 6), ln(β/T_p_^2^) and 1/T_p_ of EP/ODA-BOZ were plotted (Figure 7a), and lnβ and 1/T_p_ were plotted (Figure 7b). The average activation energy of the two prepolymers is given by the slope of the fitted lines. In addition, the determination coefficient (R^2^) of the fitted curve is calculated, and the results show that there is a good linear relationship between the heat release rate and the reciprocal of the peak heat release temperature, which indicates the validity of the calculation of the activation energy. The curing process parameters and activation energy after peak splitting are summarized in Table 1. Additionally, the curing processes of the two prepolymers were obtained using the β-T extrapolation method.

**Scheme 2:**
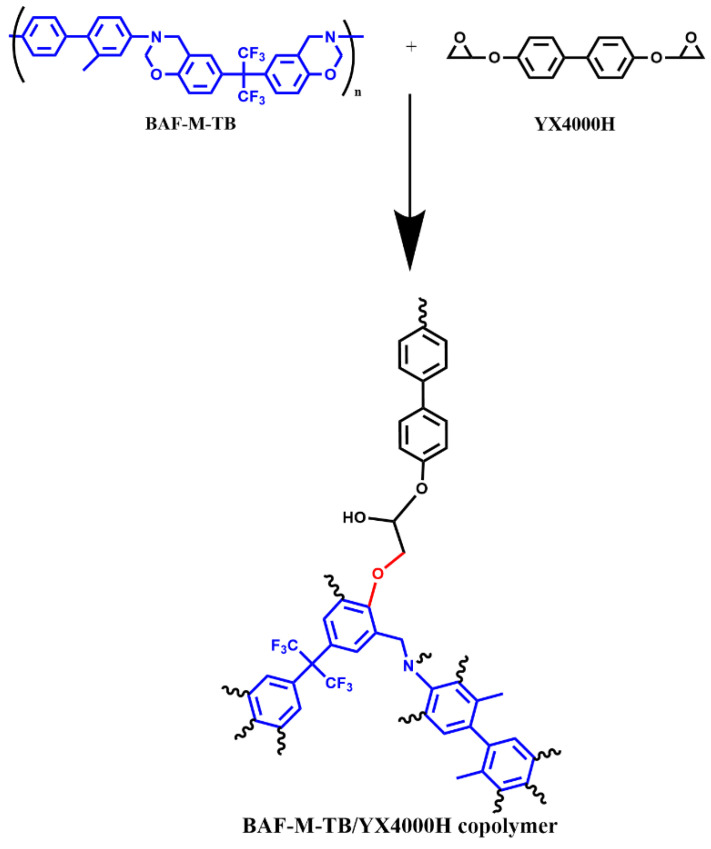
Copolymerization of YX4000 and BAF-M-TB.

To further elucidate the relationship between the characteristic peaks and structural changes in the two thermal curing stages, the curing kinetics of BAF-M-TB were also studied, and the DSC thermogram is shown in Figure S2. The average activation energy of BAF-M-TB was 109.84 kJ/mol, which is close to the average activation energy of EP/BAF-M-TB at the first exothermic peak. This indicates that the first-stage curing exothermic behavior of EP/BAF-M-TB is the self-curing behavior of BAF-M-TB, because epoxy cannot self-cure in the system. The second-stage exothermic behavior is attributed to the crosslinking of benzoxazine and epoxy, which is consistent with the conclusion obtained by Jubsilp et al. [35], and the reaction mechanism of the epoxy and BAF-M-TB is shown in Scheme 2.

**Figure 8:**
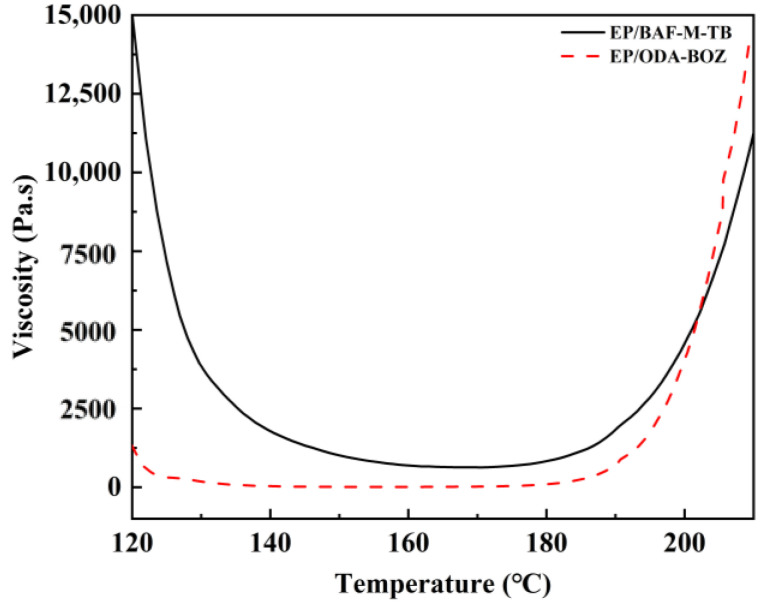
Rheological behaviors of the two prepolymers from 120 °C to 210 °C.

**Figure 9:**
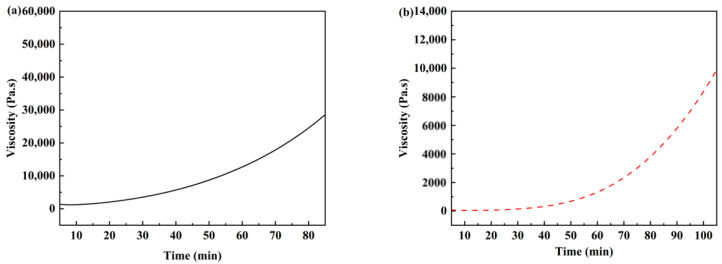
(**a**) Rheological behaviors of the EP/BAF-M-TB prepolymers at 170 °C. (**b**) Rheological behaviors of the EP/ODA-BOZ prepolymers at 150 °C.

Complex viscosity (η*) curves of the EP/BAF-M-TB prepolymer and the EP/ODA-BOZ prepolymer as a function of temperature are shown in Figure 8. The composite viscosity of the two prepolymers decreased with the increase in temperature and began to increase after a certain temperature, which corresponds to the melting and crosslinking reaction of epoxy and benzoxazine blends, separately. The lowest viscosity of EP/BAF-M-TB appears at 170 °C, and the lowest viscosity is 653.24 Pa·s. The time–viscosity curve of the EP/BAF-M-TB prepolymer at 170 °C is shown in Figure 9a, where the prepolymer melt was maintained for about 20 min in low viscosity (η < 2000 Pa·s). The lowest viscosity of EP/ODA-BOZ appears at 150 °C, and the lowest viscosity is 12.22 Pa·s. The time–viscosity curve of the EP/ODA-BOZ prepolymer at 150 °C is shown in Figure 9b, where the prepolymer melting was maintained for 67 min in low viscosity (η < 2000 Pa·s). The viscosity of the EP/BAF-M-TB system is higher, which is directly attributed to the higher molecular weight of BAF-M-TB. Both blended resin systems possess suitable machinability and processing windows, so the hot-pressing process can be utilized to make composite materials.

**Figure 10:**
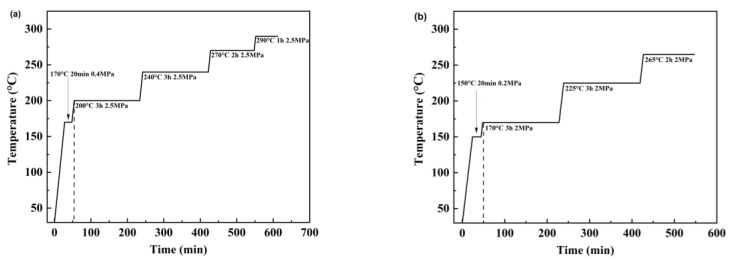
(**a**) The processing of EP/BAF-M-TB QFRP. (**b**) The processing of EP/ODA-BOZ QFRP.

Combined with the curing behavior and rheological behavior of the EP/BAF-M-TB prepolymer and the EP/ODA-BOZ prepolymer, the processing of QFRP based on the prepolymers is determined and shown in Figure 10. It is important to note that the heating rate should not be too fast during the processing procedure; otherwise, the curing speed of the surface layer will be too fast, and it cannot be well-integrated with the core layer resin, resulting in the lamination or cracking of the finished product. Therefore, first, the heat is held for a period of time under low-pressure conditions. During this period, the prepolymers in the prepreg cloth are sufficiently melted so that the multiple layers of the prepreg cloth can be fully infiltrated and bonded together. During this process, the press needs to vacuum to ensure that the air in the prepreg cloth can be successfully extruded and exhausted before heating up. After heating to the curing starting point, the system begins to crosslink, and the viscosity increases sharply, so high pressure was applied from this point until curing was completed. The EP/BAF-M-TB system is used at higher pressures during processing. This is due to the high viscosity of EP/BAF-M-TB, and the use of higher pressure can make the quartz fiber cloth and the resin bond better. However, for the EP/OA-BOZ system, the pressure is too high for a system with relatively low viscosity, and the melted resin in the prepreg cloth is easily extruded, resulting in a low glue content in the composite material, which affects the material properties.

### 3.3. Thermal Stability of Poly-EP/BAF-M-TB and Poly-EP/ODA-BOZ

**Figure 11:**
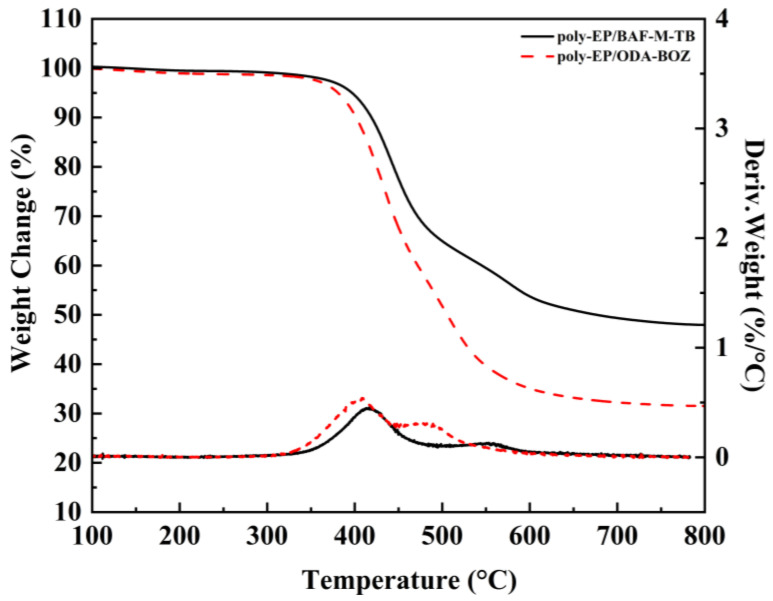
TGA curves and DTG curves of poly-EP/BAF-M-TB and poly-EP/ODA-BOZ.

The TGA thermograms of poly-EP/BAF-M-TB and poly-EP/ODA-BOZ are shown in Figure 11, and the curves of the derivative of TGA (DTG) are shown in the Figure 11. The degradation temperature at 5% (Td_5_) and 10% (Td_10_) weight loss and the char yield at 800 °C (CY8_00_) of poly-EP/F-M-TB and poly-EP/ODA-BOZ are summarized in Table 2. Compared with poly-EP/ODA-BOZ, poly-EP/BAF-M-TB clearly has higher Td_5_, Td_10_, and CY_800_ values. Both co-curing systems generate ether bonds in the cross-linked network after polymerization, and the ether bonds are easily broken by heat [36]. Compared with ODA-BOZ, BAF-M-TB has a rigid biphenyl structure and higher bond energy of trifluoromethyl, which makes EP/BAF-M-TB have higher Td_5_ and Td_10_ values. At the same time, after the ether bond is completely destroyed at high temperatures, more fragments with larger molecular weights remain in the EP/BAF-M-TB system compared with EP/ODA-BOZ, thus resulting in a higher CY_800_ value [37]. Based on the DTG curves (Figure 11), the thermal degradation process of the two QFRPs can be divided into two main parts: the first range of weight loss is located at 350–450 °C, and the second is located at 450–600 °C, approximately. The weightlessness at these two locations corresponds to the degradation of the overhang group and the release of amines and phenolic compounds via the breaking of the main-chain Mannich bridge, respectively [38,39].

### 3.4. Thermal and Mechanical Properties of Poly-EP/BAF-M-TB QFRP and Poly-EP/ODA-BOZ QFRP

**Figure 12:**
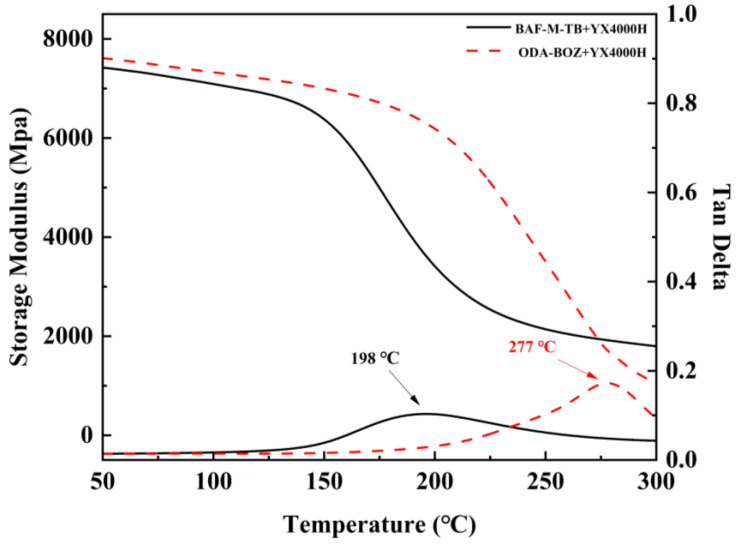
DMA thermograms of poly-EP/BAF-M-TB QFRP and poly-EP/ODA-BOZ QFRP.

The DMA thermograms of poly-EP/BAF-M-TB QFRP and poly-EP/ODA-BOZ QFRP are shown in Figure 12. The thermograms show that poly-EP/ODA-BOZ QFRPs have a larger storage modulus (E’) than poly-EP/BAF-M-TB QFRPs at 50 °C. Meanwhile, the T_g_ values determined by the tan δ peaks of the poly-EP/BAF-M-TB QFRP and the poly-EP/ODA-BOZ QFRP are 198 °C and 277 °C, respectively. Both composites showed high rigidity and T_g_, while poly-EP/ODA-BOZ QFRP showed better performance. BAF-M-TB with biphenyl groups and a higher molecular weight has lower degrees of freedom when forming cross-linked networks, so it tends to form a more regular structure than ODA-BOZ, which is conducive to improving the rigidity and T_g_ of the polymer [19]. However, due to the large molecular volume of BAF-M-TB materials, epoxy cannot completely react with them and only parts of them can self-cure, while ODA-BOZ can react well with epoxy; this finding is consistent with the above study on the curing behavior of the two prepolymers. In addition, trifluoromethyl has high spatial resistance, which also makes the cross-linking network of poly-EP/BAF-M-TB looser than that of poly-EP/ODA-BOZ [29].

**Figure 13:**
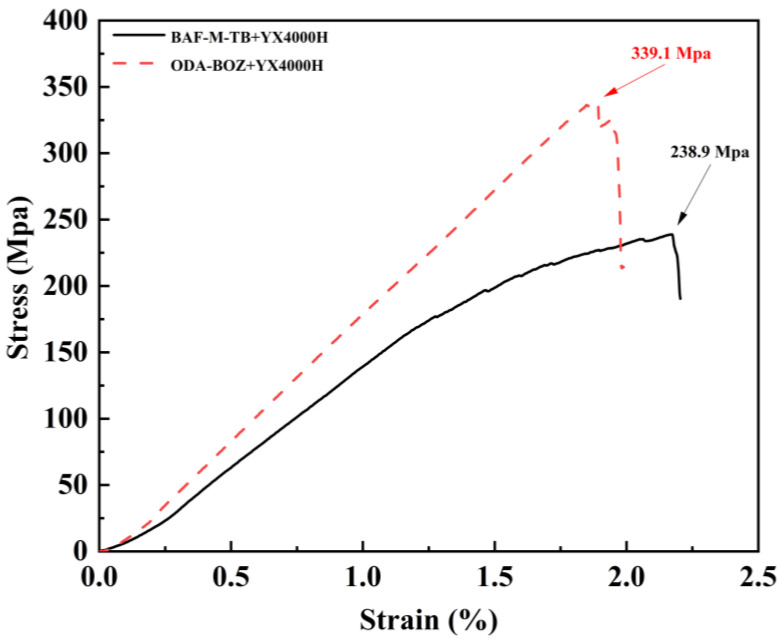
Stress–strain curves of poly-EP/BAF-M-TB QFRP and poly-EP/ODA-BOZ QFRP.

The bending properties of poly-EP/BAF-M-TB QFRP and poly-EP/ODA-BOZ QFRP were measured, and the results are shown in Figure 13. Both composites showed high toughness, and poly-EP/ODA-BOZ QFRP showed higher flexural strength, which is attributed to the higher cross-link density of EP/ODA-BOZ. In addition, poly-EP/BAF-M-TB QFRP exhibits higher flexural strain at break, which is attributed to the higher free volume in EP/BAF-M-TB [12].

### 3.5. Dielectric Properties of Poly-EP/BAF-M-TB QFRP and Poly-EP/ODA-BOZ QFRP

**Figure 14:**
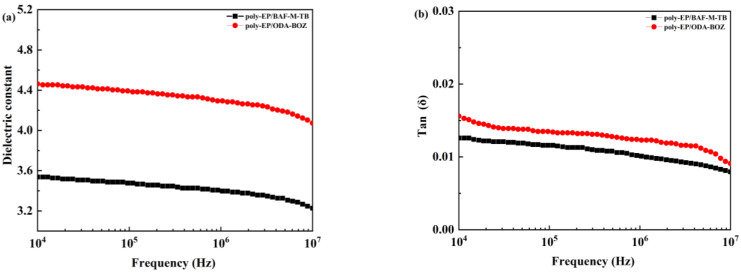
(**a**) The dielectric constant of the poly-EP/BAF-M-TB QFRP and the poly-EP/ODA-BOZ QFRP at low frequencies. (**b**) The dielectric loss of the poly-EP/BAF-M-TB QFRP and the poly-EP/ODA-BOZ QFRP at low frequencies.

**Scheme 3:**
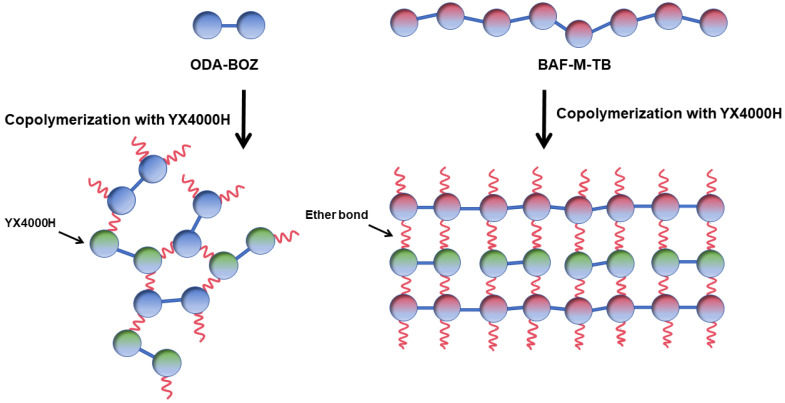
Illustration of the crosslink network of EP/ODA-BOZ and EP/BAF-M-TB.

The low-frequency dielectric constant (k) and dielectric loss (f) of poly-EP/BAF-M-TB QFRP and poly-EP/ODA-BOZ QFRP are shown in Figure 14. The high-frequency dielectric constant (k) and dielectric loss (f) values of the poly-EP/BAF-M-TB QFRP and poly-EP/ODA-BOZ QFRP are presented in Table 3. The poly-EP/BAF-M-TB QFRP exhibits a lower dielectric constant and dielectric losses than the poly-EP/ODA-BOZ QFRP at low frequencies, and a lower dielectric constant at high frequencies. However, the dielectric loss is slightly higher at a high frequency. The lower dielectric constant is attributed to the C-F bond in poly-EP/BAF-M-TB QFRP, which is difficult to polarize; this can effectively reduce the polarizability of molecules, thus improving the dielectric properties. The biphenyl group in the main chain reduces the tight packing of molecular chains, and the free volume of the polymer is increased. In addition, the crosslinking networks of EP/ODA-BOZ and EP/BAF-M-TB are illustrated in Scheme 3. Compared with EP/OAD-BOZ, EP/BAF-M-TB has a more structured cross-linking network, which helps the alcohol hydroxyl groups formed by copolymerization to arrange regularly and form intermolecular hydrogen bonds, thus reducing the polarity of the copolymerization system [22]. The dielectric loss is related to the polarization density and crosslinking density of the polymer network. At low frequencies, the dielectric loss of poly-EP/BAF-M-TB QFRP is significantly better than that of poly-EP/ODA-BOZ QFRP due to the presence of trifluoromethyl. However, the polarization of the dipole does not occur at high frequencies, the dielectric loss factor is more sensitive to the crosslinking density, and the dielectric loss will gradually decrease with the increase in the crosslinking density [40,41].

## 4. Conclusions

In this study, a new fluorine-containing main-chain benzoxazine precursor (BAF-M-TB) was synthesized. The molecular structure of the precursor was verified using FTIR, NMRs, and SEC. ODA-BOZ, a benzoxazine commonly used in commercial industries, was used as a control. Two kinds of benzoxazines were blended with epoxy. The thermal curing behaviors, curing kinetics, and rheological behavior of EP/BAF-M-TB and EP/ODA-BOZ were studied. The results show that the two blending systems meet the requirements for processing QFRP composites, and the process flow for preparing QFRPs via the hot-pressing method was obtained. Both composites showed good thermal properties. Compared with the blend of epoxy and ODA-BOZ, poly-EP/BAF-M-TB showed better thermal stability, and the cured poly-EP/BAF-M-TB QFRP had better dielectric properties at both low and high frequencies.

## Figures and Tables

**Table 1:** The curing process parameters and curing kinetic parameters of EP/BAF-M-TB and EP/ODA-BOZ.

Curing Behavior	Heating Rate (°C/min)	T_i_ (°C)	T_p_ (°C)	T_d_ (°C)	E_a_ (kJ/mol) (Kissinger Method)	E_a_ (kJ/mol) (Ozawa Method)	E_a_ (kJ/mol) (Average)
BAF-M-TB self-polymerization	5	207.1	243.8	281.7	103	106.3	104.65
	10	220.3	260.9	294.3			
	15	236	269.1	300			
	20	229	270.8	307.4			
EP/BAF-M-TB copolymerization	5	251.3	283.3	313.3	189.6	189.1	189.35
	10	259	291	322.3			
	15	271	296.3	327			
	20	283.3	302.1	318			
EP/ODA-BOZ copolymerization	5	175	229	271.3	78.3	82.6	80.45
	10	184.5	244.7	287			
	15	188.3	253.3	296.8			
	20	193.3	265.7	301			

**Table 2:** Thermal stability of poly-EP/BAF-M-TB and poly-EP/ODA-BOZ.

Sample	T_d5_ (°C)	Td_10_ (°C)	CY_800_ (%)
Poly-EP/BAF-M-TB	398	420	47.95
Poly-EP/ODA-BOZ	380	401	31.50

**Table 3:** The dielectric constant and dielectric loss of the poly-EP/BAF-M-TB QFRP and the poly-EP/ODA-BOZ QFRP at high frequencies.

Sample	Dielectric Constant at 5 GHz	Dielectric Loss at 5 GHz
Poly-EP/BAF-M-TB	3.16	0.016
Poly-EP/ODA-BOZ	4.01	0.012

## Data Availability

Data available upon request due to ethical or privacy-related restrictions. The data presented in this study are available upon request from the corresponding author. The material and its homologues are currently being extensively studied by us for potential industrialization, and the data cannot be directly disclosed.

## Supplementary Materials

The following supporting information can be downloaded at: https://www.mdpi.com/article/10.3390/polym15234487/s1, Figure S1: SEC result of BAF-M-TB. Figure S2: DSC thermograms of BAF-M-TB.
