# Supplementary material for: A Fluorine-Containing Main-Chain Benzoxazine/Epoxy Co-Curing System with Low Dielectric Constants and High Thermal Stability
Source: Polymers (Basel). 2023 Nov 22;15(23):4487. doi: 10.3390/polym15234487 (PMC10707784; doi:10.3390/polym15234487)
Supplement: Supplementary file 1 [file polymers-15-04487-s001.zip › polymers-2678172-supplementary.pdf]

polymers

Cirrus GPC Sample Peak Report

Generated by: dell 2023 / 10 / 23 14:54
Workbook: D:\Cirrus Workbooks\20220121-RI ONLY\20220121-RI ONLY.plw

Sample Details

Sample Name: ZTH	
Acquired: 2023/8/6 14:20:10	By Analyst: dell
Batch Name: 20230806	
Filename: D:\Cirrus Workbooks\20220121-RI ONLY\20230806-0001.cgrm	
Concentration: 0.10 mg/ml	K of Sample: 14.1000
Injection Volume: 100.0 ul	Alpha of Sample: 0.7000
LIMS ID:	Bottle ID:

Workbook Details

Eluent: NMP	Flow Rate: 1.00 ml/min
Column Set:	Column Set Length: 650 mm
Detector: RI	Temperature: 80

Analysis Using Method: 20221001

Comments:

Results File: D:\Cirrus Workbooks\20220121-RI ONLY\20230806-0001-Repeat (01).rst

Calibration Used: 2022/11/24 16:04:14

Calibration Type: Narrow Standard	Curve Fit Used: 1
Calibration Curve: y = 12.680313 - 0.546750x^1	
High Limit MW RT: 10.78 mins	Low Limit MW RT: 18.29 mins
High Limit MW: 6088701	Low Limit MW: 480
K: 14.1000	FRM Name:
Alpha: 0.7000	Flow Marker RT: 23.30 mins
FRCF: 1.0000	

MW Averages

Mp: 7544	Mn: 4540	Mv: 10799	Mw: 12797
Mz: 43341	Mz+1: 118859	PD: 2.8187	

Distribution Plots

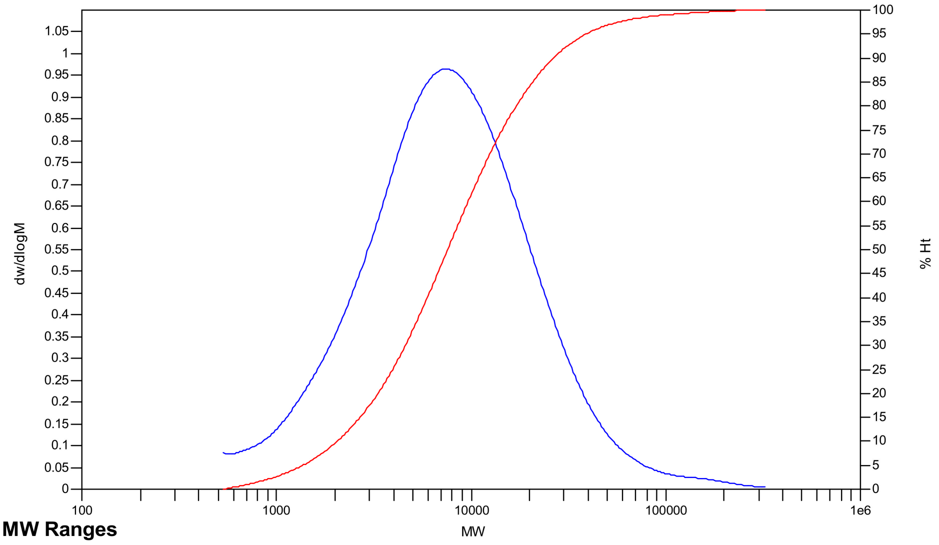

MW Ranges

Figure S1. SEC result of BAF-M-TB.

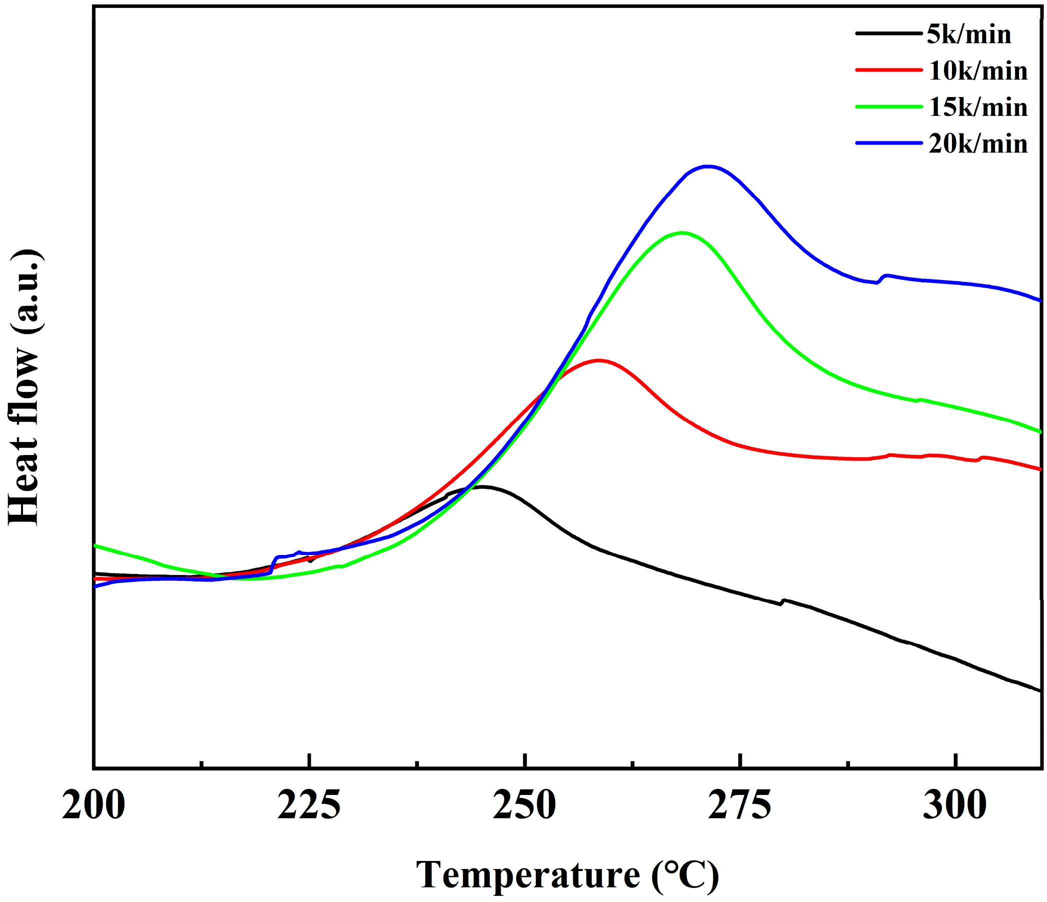

Figure S2. DSC thermograms of BAF-M-TB.
